# Supplementary material for: Identification of photoperiod-induced specific miRNAs in the adrenal glands of Sunite sheep (Ovis aries)
Source: Front Vet Sci. 2022 Jul 22;9:888207. doi: 10.3389/fvets.2022.888207 (PMC9354845; doi:10.3389/fvets.2022.888207)
Supplement: Supplementary file 1 [file Data_Sheet_1.ZIP › Supplementary file/miRDeep2 file.zip!/miRDeep2 file/Know_Structure/LP42A1/oar-mir-409.pdf]

miRBase precursor : oar-mir-409
Total read count : 12018
oar-miR-409-5p read count : 2297
oar-miR-409-3p read count : 9714
remaining reads : 7

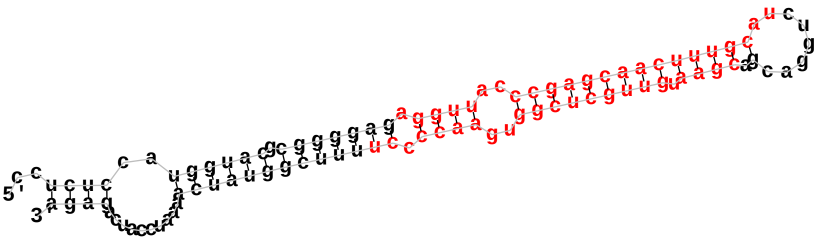

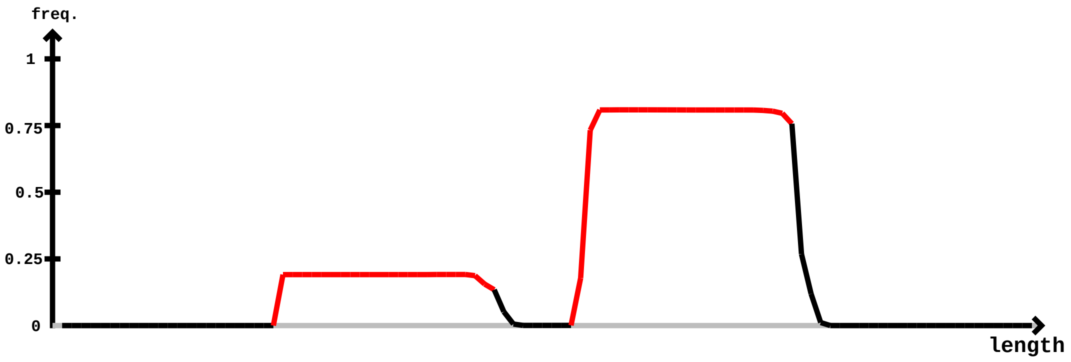


```
                                     oar-miR-409-5p
                                                                oar-miR-409-3p
5'- ccucuccaugguacgcggggagagguuacccgagcaacuuugcaucuggacgacgaauguugcucggugaaccccuuuucgguaucaaaauccaucagaga   -3'    exp
    .((((...((((((.(((((((.((((..(((((((((((((........).)))).)))))))))..)))).)))))))))))))........)))).     reads      mm         sample
    ......................agguuacccgagcaacuuu............................................................   2          0          seq
    ......................agguuacccgagcaacuuug...........................................................   54         0          seq
    ......................agguuacccgagcaacuuugc..........................................................   376        0          seq
    ......................agguuacccgagcaacuuugca.........................................................   256        0          seq
    ......................agguuacccgagcaacuuugcau........................................................   990        0          seq
    ......................agguuacccgagcaacuuugcauc.......................................................   555        0          seq
    ......................agguuacccgagcaacuuugcaucu......................................................   62         0          seq
    .......................gguuacccgagcaacuuugc..........................................................   2          0          seq
    ......................................uuugcaucuggacgacgaauguu........................................   1          0          seq
    ......................................uuugcaucuggacgacgaauguug.......................................   1          0          seq
    ......................................uuugcaucuggacgacgaauguugc......................................   1          0          seq
    ......................................uuugcaucuggacgacgaauguugcu.....................................   1          0          seq
    ........................................ugcaucuggacgacgaauguugcu.....................................   1          0          seq
    .........................................gcaucuggacgacgaauguugcu.....................................   1          0          seq
    ..........................................caucuggacgacgaauguugcuc....................................   1          0          seq
    ....................................................acgaauguugcucggugaac.............................   2          0          seq
    .....................................................cgaauguugcucggugaa..............................   1          0          seq
    .....................................................cgaauguugcucggugaac.............................   11         0          seq
    .....................................................cgaauguugcucggugaacc............................   24         0          seq
    .....................................................cgaauguugcucggugaaccc...........................   44         0          seq
    .....................................................cgaauguugcucggugaacccc..........................   170        0          seq
    .....................................................cgaauguugcucggugaaccccu.........................   1359       0          seq
    .....................................................cgaauguugcucggugaaccccuu........................   366        0          seq
    .....................................................cgaauguugcucggugaaccccuuu.......................   136        0          seq
    .....................................................cgaauguugcucggugaaccccuuuu......................   19         0          seq
    .....................................................cgaauguugcucggugaaccccuuuuc.....................   1          0          seq
    ......................................................gaauguugcucggugaac.............................   4          0          seq
    ......................................................gaauguugcucggugaacc............................   8          0          seq
    ......................................................gaauguugcucggugaaccc...........................   46         0          seq
    ......................................................gaauguugcucggugaacccc..........................   270        0          seq
    ......................................................gaauguugcucggugaaccccu.........................   3890       0          seq
    ......................................................gaauguugcucggugaaccccuu........................   1300       0          seq
    ......................................................gaauguugcucggugaaccccuuu.......................   1050       0          seq
    ......................................................gaauguugcucggugaaccccuuuu......................   98         0          seq
```

```
                      oar-miR-409-5p
                                                     oar-miR-409-3p
ccucuccaugguacgcggggagagguuacccgagcaacuuugcaucuggacgacgaauguugcucggugaaccccuuuucgguaucaaaauccaucagaga

.......................................................aauguugcucggugaacc............................    1     0     seq
.......................................................aauguugcucggugaaccc...........................    8     0     seq
.......................................................aauguugcucggugaacccc..........................    28    0     seq
.......................................................aauguugcucggugaaccccu.........................    626   0     seq
.......................................................aauguugcucggugaaccccuu........................    124   0     seq
.......................................................aauguugcucggugaaccccuuu.......................    107   0     seq
.......................................................aauguugcucggugaaccccuuuu......................    17    0     seq
........................................................auguugcucggugaaccccuuu.......................    1     0     seq
.........................................................uguugcucggugaaccccuu........................    1     0     seq
.........................................................uguugcucggugaaccccuuu.......................    1     0     seq
.........................................................uguugcucggugaaccccuuuu......................    1     0     seq
```
